# Self-awareness for financial decision-making abilities in healthy adults

**DOI:** 10.1371/journal.pone.0235558

**Published:** 2020-07-02

**Authors:** Preeti Sunderaraman, Silvia Chapman, Megan S. Barker, Stephanie Cosentino

**Affiliations:** 1 Cognitive Neuroscience Division of the Taub Institute for Research on Alzheimer’s Disease and the Aging Brain, Columbia University Medical Center, New York, NY, United States of America; 2 Gertrude. H. Sergievsky Center, Columbia University Medical Center, New York, NY, United States of America; 3 Department of Neurology, Columbia University Medical Center, New York, NY, United States of America; University of Texas Southwestern Medical Center at Dallas, UNITED STATES

## Abstract

**Objective:**

Decades of research have established how to measure metacognition (i.e., awareness of one’s cognitive abilities), whereas relatively little is known about how to assess the integrity of financial awareness (FA; awareness of one’s financial abilities), a related construct with practical implications for vulnerable older adults. The current study’s goal was to apply established metacognitive frameworks to identify an objective measure of FA.

**Methods:**

Metacognitive ratings were integrated into two financial decision making (FDM) assessments in order to derive two types of FA metrics: absolute accuracy (calibration) and relative accuracy (resolution) in each FDM task. Associations between each FA metric, demographic variables, FDM performances, and metamemory were examined.

**Design & setting:**

Cross-sectional, community-based, prospective study.

**Participants:**

93 individuals with mean age = 59 years (SD = 15.12); mean education = 15.70 (SD = 2.39); 60% females.

**Measures:**

FA was calculated using the Financial Competency Assessment Inventory (FCAI) and Decision Making Competence Assessment Tool, Finance Module (DMC-F), and memory awareness was calculated using an objective metamemory test.

**Results:**

None of the FA metrics was associated with age, education or gender. FCAI calibration was inversely associated with FDM, and positively correlated with DMC-F calibration and metamemory calibration. None of the FA metrics for DMC-F was associated with metamemory.

**Conclusions:**

Mirroring findings from metamemory studies, overconfidence in FDM was associated with lower FDM accuracy in healthy adults. Moreover, calibration scores on the FCAI and metamemory were related, suggesting that FA taps into metacognitive abilities. Our findings provide preliminary evidence for how to measure FA in both clinical and research contexts.

## Introduction

Self-awareness, an individual’s knowledge of a single or multiple aspect(s) of the self, has fascinated scientists and philosophers for centuries [1]. In recent years, researchers have shown that self-awareness has critical implications for everyday functioning and decision making [2–5]. Against this backdrop, financial awareness (FA), or the ability to gauge one’s own financial decision-making (FDM) abilities, is a novel, emerging construct that has gained importance with the increased focus on financial wellness and longevity fitness [6]. In a recent, longitudinal study of 9,434 older adults aged 65 and above, about 30% endorsed difficulty managing finances over a 10-year period [7]. The extent to which older adults are aware of and can navigate or compensate for such difficulties, however, is unknown.

Decreased FA likely puts individuals of any age at risk for incurring financial loss through various mechanisms such as falling victim to scams, overspending, compulsive spending, or via making poor investment decisions, and therefore has significant clinical and public policy implications [2, 8–10]. It is thus integral to include FA in the assessment of FDM, and to monitor this type of awareness longitudinally as a marker of independent functioning. Before studying if and how FA deteriorates and/or serves as a risk factor for financial loss across the lifespan, or in the context of pathologic aging, it is critical to first determine how to operationalize FA.

Research into FA has thus far been limited, and the manner in which FA should be measured is not yet clear. Like other studies examining awareness of cognitive abilities, FA has thus far been measured as the discrepancy between a participant’s evaluation of their abilities and that of a knowledgeable informant [6, 9, 11, 12]. From a practical standpoint, gathering data from a caregiver is common and becomes necessary for those individuals whose self-report about their everyday functioning may be suspicious or even unreliable, e.g., in those with memory deficits [13, 14]. However, relying on informant report as the gold standard is problematic and not ideal because the caregiver may not be knowledgeable enough about the individual or may even be biased due to other factors such as caregiver burden [13, 15]. As a result, the assessment of self-awareness for cognitive abilities has moved toward incorporating objective measures developed in the field of cognitive psychology to study metacognition (i.e., knowing about knowing) [16]. The current study evaluates the utility of two established metacognitive scoring approaches, gathered through objective assessment, to optimally characterize FA.

Typically, metacognitive tasks evaluate an individual’s knowledge about their cognitive abilities by ascertaining judgments regarding ongoing task performance and measuring such judgments against task performance. The accuracy of such judgments is most typically examined via two primary metrics: absolute accuracy (*calibration*) and relative accuracy (*resolution*) [17–20]. Whereas resolution reflects the extent to which predictions are adjusted in accord with shifts in performance on each item of the task, calibration represents the average degree of over- or under-confidence regarding task performance. In everyday life, poor resolution would translate to individuals not knowing which specific aspects of FDM they are good at, and which they are bad at. For example, they may think that they have a good grasp of financial terms and concepts, but in reality have a fairly shallow grasp of these terms. Or, they may feel that they are good at selecting insurance plans, but in actuality they may be selecting plans with high deductibles and poor coverage. Calibration would reflect individuals' overall level of confidence regarding financial decision making in general, as a whole. It is possible, for example, that individuals may be well calibrated overall in their sense of having average FDM abilities, but have poor resolution regarding which tasks they are good at and which they are bad at. In contrast, they may have a good sense of which tasks they are better or worse at (resolution), but be highly over or under confident on the whole. These two awareness metrics are independent of one another; for example, an individual can have good resolution but be overconfident on the whole, or vice versa, can be perfectly calibrated but make predictions that do not track with performance on an item-by-item basis. The utility of each of these metrics for quantifying and understanding FA needs to be evaluated directly.

Specifically, the utility of these metrics for quantifying FA can be assessed in several ways. First, it is important to understand the manner in which these metrics relate to FDM itself. Awareness of an ability (e.g., metamemory) can become quite dissociated from the ability itself (e.g., memory) in the context of brain injury [16, 19], but in cognitively intact individuals, metacognition and cognition are often closely linked, such that individuals with higher metacognitive scores (e.g., metamemory) perform better on corresponding cognitive tasks (e.g., memory) [21]. In addition, more accurate self-assessment in one domain appears to be associated with more accurate self-assessment in another [22]. As such, among cognitively healthy adults, it is reasonable to expect that FA should be related to FDM, and that awareness across different FDM tasks and metamemory should be associated with each other. The current study examined each type of FA score (i.e., calibration and resolution) in reference to three criteria. Specifically, we posit that the optimal FA metric will have an at least medium to large effect size when examining it’s association with: (1) FDM itself (i.e., task accuracy), (2) the corresponding FA metric on a separate FDM task, and (3) performance on a metamemory task, a validated metacognitive metric that captures a different aspect of self-awareness.

## Materials and methods

### Participants

Data were prospectively collected from 93 cognitively healthy adults (60 older and 33 young/middle-aged henceforth referred to as “younger”). All older participants and 12 younger participants were recruited from ongoing studies of cognitive aging while the remaining 21 younger participants were recruited from the community through flyers. Participants were required to be native English speakers, have a minimum of fourth-grade reading level, not be diagnosed with a neurological condition, not have a recent, unmanaged psychiatric condition, and not have hearing and visual impairment that would interfere with testing. Participants drawn from ongoing studies were screened for dementia or MCI using the Dementia Rating Scale (DRS; cutoff = 135). The younger participants recruited from the community were screened using the Mini Mental Status Examination (cutoff = 27). The Internal Review Board of the College of Physicians and Surgeons of Columbia University approved this study. Prior to the testing session, written informed consent was obtained from all participants and compensation was provided at the end of the study.

## Measures

### Metamemory test

This task consisted of four trials with five items in each trial, yielding a total of 20 metamemory items. Briefly, participants are taught five pieces of “pseudo trivia” across four learning trials, about an individual from history paired with fake information about the individual’s background (e.g., “Cole Porter attended law school in Chicago”; for the full task description see [21, 23, 24]. Performance accuracy was dichotomous (correct or incorrect) for each item. Before each item, participants were asked to predict if they would recognize the correct answer from among eight answer choices (Yes, Maybe, No). These ordinal ratings were converted to interval data (1, 0.5, and 0). To obtain calibration scores, average accuracy was subtracted from the average prediction score to determine the extent to which individuals were over- or underconfident on an item-by-item basis. A score of zero indicated perfect calibration, positive scores indicated overconfidence, and negative scores indicated underconfidence (total score range: -1 to 1).

To obtain resolution scores, the nonparametric Goodman-Kruskal gamma statistic, a rank order correlation was employed [25]. Although we included only the prospective ratings to calculate resolution scores, data suggests that amongst healthy older adults, prospective and retrospective ratings are comparable [4, 22]. Higher gamma scores reflected better resolution or better ability to predict performance (total score range: -1 to 1).

### Decision Making Competence Assessment Tool, Finance Module (DMC-F)

This task consisted of 12 items measuring financial and healthcare decision-making [26]. The finance module, which consisted of 6 items, was included in the current analysis [27]. The items consist of tables providing mutual funds information in which participants had to select the correct fund based on a set of a priori criteria. The items were structured to increase in difficulty level. Performance accuracy is dichotomous (correct or incorrect) and ranged from 0 to 6. After administering each item, participants made retrospective judgments about their performance on the specified item. Per test instructions, confidence ranged on a scale from 1 (not at all confident) to 4 (very confident). As per the original task structure, the accuracy and confidence levels consisted of different ranges, and only retrospective ratings are collected, At the time of data collection, we retained the original structure of the task to keep it consistent with previous studies [27]. A basic premise underlying the calculation of calibration scores is that accuracy and confidence scores have the same range. Therefore, to bring the accuracy and confidence ratings onto the same scale, confidence ratings were recoded on a 3-point scale as 0, 0.5, and 1 (see S1 Data). Calibration (total score range: -1 to 1) and resolution (total score range: -1 to 1) scores were calculated as described for the metamemory test.

### Financial Competence Assessment Inventory (FCAI)

The original FCAI consists of 38 self-report and performance-based items [28]. For the purposes of the current study, 20 objective items were selected [13]. The items were either performance-based and observable (e.g., writing a check) or were conceptual knowledge questions that could be scored objectively with an external criterion (e.g., what is the meaning of assets?). Accuracy was originally scored on a scale from 1 to 5, and for the purpose of the current analysis it was collapsed into 1 to 3, with total accuracy ranging from 20 to 60. Before and after each item, participants made prospective and retrospective judgments about their performance on the specified item on a scale from 1 (not at all confident) to 4 (very confident). As described for the DMC-F, the original scales were collapsed into a 3-point scale ranging from 1 to 3 (see S1 Data). Modeled after the Metamemory task, calibration (total score range: -2 to 2) and resolution (total score range: -1 to 1) scores were calculated separately for both the predictions and post-dictions.

## Procedures

Participants completed a measure of global cognition (either Dementia Rating Scale; DRS or Mini Mental State Examination; MMSE) prior to study participation. All the three tasks were administered in a single session. The metamemory task was given first, while the DMC-F and FCAI were then administered in a counterbalanced manner. The participants were given $40 at the end of the study session and money to cover the travel expenses.

## Data analysis

Analyses were performed using Predictive Analytics SoftWare (PASW) 22.0 and JASP 0.11.1. Non-parametric statistics were used given the distribution of data and the violations of normality assumptions. Bonferroni corrections were used to adjust for multiple comparisons and effect sizes using Cohen’s guidelines (1992) were used to interpret the results [29].

We first calculated Spearman’s correlations between FDM accuracy, FA metrics (calibration and resolution) and demographics (age, education and sex). In the absence of significant age-group differences, we combined the data to increase power. Finally, we obtained correlations within and across performance accuracy, calibration and resolution for the Metamemory, FCAI, and DMC-F tasks.

## Results

Descriptive statistics for demographics, performance accuracy and awareness metrics are provided in Table 1. Internal consistency (α) for the accuracy of the 3 measures was .83 for Metamemory, .71 for FCAI, and .44 for DMC-F. No significant differences in education, gender, or ethnicity were found between younger and older adults. Among the older adults there was a higher proportion of Caucasians than in the younger adult group, which included more African American participants and individuals from other races.

**Table 1:** Demographics, FDM accuracy, and awareness metrics for the overall sample, and split by age.

	n	Overall	Older adults	Younger adults	*t\chi* (*p*)
		Mean (SD; range)/Frequency			
**Demographics**					
Age		59.02 (15.12; 30–84)	68.95 (5.48; 60–84)	40.70 (9.02; 30–56)	18.63 (< .001)
Education (yrs)		15.70 (2.39; 7–20)	15.67 (2.28; 10–20)	15.77 (2.62; 7–20)	-0.19 0.85)
Female; n (%)		56 (60)	37 (62)	19 (58)	0.15 (0.70)
Race; n (%)					12.43 (< .001)
	Caucasians	51 (55)	41 (68)	10 (31)	
	Blacks	35 (38)	18 (30)	17 (51)	
	Asians	3 (3)	0 (0)	3 (9)	
	Native Hawaiian/ Pacific Islander	1 (1)	1 (2)	0 (0)	
	Other	3 (3)	0 (0)	3 (9)	
Ethnicity; n (%)					
	Non-Hispanic	84 (90)	55 (92)	29 (88)	0.35 (0.55)
**Performance Accuracy**^†^					
	N^‡^				
Metamemory	90	13.16 (4.33; 3–20)	13.01 (4.47; 3.5–20)	13.47 (4.09; 3–20)	
FCAI	91	53.30 (4.63; 40–60)	54 (4.44; 40–60)	52 (4.77; 40–60)	
DMC-F	91	3.87 (1.20; 0–6)	3.90 (1.16; 0–6)	3.81 (1.31; 0–6)	
**Calibration**^†^^,^^§^					
Metamemory	90	0.08 (0.16; -0.4–0.5)	0.06 (0.16; -0.4–0.43)	0.12 (0.16; -0.18–0.5)	
FCAI.pre	83	-0.07 (0.34; -1.4–0.9)	-0.04 (0.36; -1.4–0.9)	-0.12 (0.31; -0.9–0.4)	
FCAI.post	81	0.01 (0.35; -1.2–0.85)	0.04 (0.36; -1.2–0.85)	-0.04 (0.35; -1.05–0.55)	
DMC-F	90	1.12 (0.21; -0.42–0.75)	0.11 (0.21; -0.42–0.75)	0.14 (0.21; -0.33–0.58)	
**Resolution**^**∫**^					
Metamemory	79	0.63 (0.50)	0.64 (0.49)	0.61 (0.52)	
FCAI.pre	77	0.35 (0.65)	0.28 (0.71)	0.46(0.53)	
FCAI.post	73	0.39 (0.63)	0.31 (0.72)	0.53 (0.42)	
DMC-F	69	0.69 (0.63)	0.67 (0.68)	0.73 (0.52)	

^‡^Data is unequal across accuracy, calibration, and resolution variables because of missing data resulting from either invalid performance, administrative errors or a high number of ties resulting in scores not being calculated (in case of gamma).

^†^Comparison statistics provided in Table 2.

^§^Calibration ranges for Metamemory and DMC-F are -1 to 1, whereas for FCAI it is -2 to 2. Resolution for all the tasks ranges from -1 to 1.

### Association between demographics and FA metrics

After adjusting for multiple comparisons using Bonferroni corrections, age, education and gender were not significantly associated with either calibration or resolution (see Table 2). Education and gender was associated with performance accuracy, and these variables were used as covariates when conducting the correlational analysis among the accuracy metrics (see Table in S1 Text).

**Table 2:** Association between education and gender with FA calibration and resolution.

	Age	Education	Gender	
	Spearman's rho (*p*)			
**Accuracy**				
Memory	-0.12 (*0*.*28*)	.35 (.001)	.27 (.01)
FCAI	0.13 (*0*.*22*)	.51 (< .001)	.08 (.40)
DMC-F	-0.07 (*0*.*54*)	.22 (.04)	.06 (.56)
**Calibration**				
Metamemory	-0.11 (*0*.*32*)	-0.14 (*0*.*21*)	0.08 (*0*.*46*)
FCAI.pre	0.10 (*0*.*37*)	-0.16 (*0*.*16*)	-0.22 (*0*.*05*)
FCAI.post	0.12 (*0*.*27*)	-0.27 (*0*.*02*)	-0.15 (*0*.*19*)
DMC-F	-0.13 (*0*.*24*)	-0.02 (*0*.*89*)	0.14 (*0*.*18*)
**Resolution**				
Metamemory	0.13 (*0*.*24*)	-0.02 (*0*.*82*)	0.18 (*0*.*12*)
FCAI.pre	-0.09 (*0*.*41*)	0.11 (*0*.*35*)	0.03 (*0*.*79*)
FCAI.post	-0.01 (*0*.*93*)	0.09 (*0*.*47*)	0.02 (*0*.*88*)
DMC-F	0.09 (*0*.*45*)	0.12 (*0*.*32*)	-0.01 (*0*.*93*)

### Associations between FDM, FA, and metamemory (See Table 3)

#### I. Accuracy

Accuracy scores on all 3 tasks were intercorrelated, with the association between the two financial tasks being higher than that with memory accuracy. The effect size was medium for the associations between the financial tasks (r = .445) and was in the small to medium range for the associations between financial tasks and memory accuracy (r range = .260-.345).

**Table 3:** Association between FDM, FA, and metamemory.

			1		2		3		4		5		6		7	8	9		10	11
	**Performance Accuracy**																			
1	Memory score from Metamemory Test	Spearman's rho	—																	
		p-value	—																	
2	FCAI	Spearman's rho	0.345	***	—															
		p-value	< .001		—															
3	DMC-F	Spearman's rho	0.260	*	0.445	***	—													
		p-value	0.014		< .001		—													
	**Calibration**																			
4	Metamemory	Spearman's rho	-0.586	***	-0.222	*	-0.136		—											
		p-value	< .001		0.038		0.206		—											
5	FCAI.pre	Spearman's rho	-0.321	**	-0.484	***	-0.314	**	0.317	**	—									
		p-value	0.003		< .001		0.004		0.004		—									
6	FCAI.post	Spearman's rho	-0.391	***	-0.609	***	-0.378	***	0.359	**	0.886	***	—							
		p-value	< .001		< .001		< .001		0.001		< .001		—							
7	DMC-F	Spearman's rho	-0.037		-0.155		-0.459	***	0.051		0.298	**	0.354	**	—					
		p-value	0.736		0.150		< .001		0.641		0.007		0.001		—					
	**Resolution**																			
	Metamemory	Spearman's rho	0.330	**	0.193		-0.065		-0.195		-0.079		-0.205		-0.074	—				
8		p-value	0.003		0.092		0.575		0.085		0.511		0.093		0.523	—				
	FCAI.pre	Spearman's rho	0.011		-0.162		0.076		-0.007		-0.055		-0.038		0.016	-0.053	—			
9		p-value	0.922		0.159		0.519		0.950		0.635		0.750		0.895	0.668	—			
	FCAI.post	Spearman's rho	0.115		0.042		-0.007		-0.037		0.020		0.014		0.042	-0.024	0.639	***	—	
10		p-value	0.340		0.727		0.954		0.762		0.869		0.907		0.730	0.851	< .001		—	
	DMC-F	Spearman's rho	0.154		0.036		0.068		-0.213		-0.032		-0.062		-0.057	0.096	-0.043		-0.047	—
11		p-value	0.217		0.773		0.576		0.086		0.804		0.639		0.644	0.464	0.748		0.732	—

* p < .05,

** p < .01,

*** p < .001; Cohen’s effect sizes: .10-.29 = small, .30-.50 = medium, >.50 = large.

#### II. Accuracy and awareness within task

**Fig 1:**
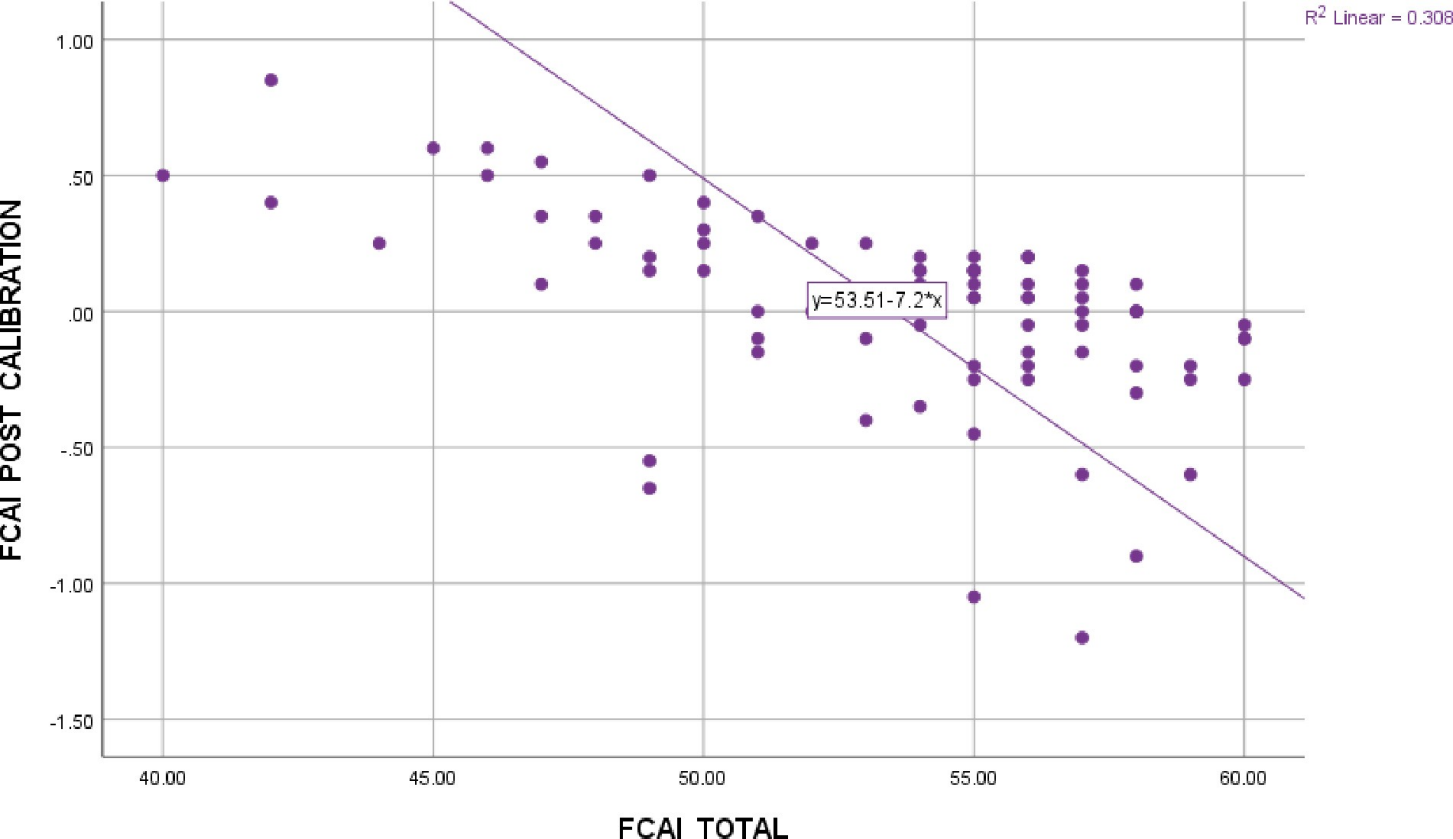


For all 3 tasks, accuracy was negatively associated with calibration, with medium to large effect sizes (see Table 3; see Fig 1). Metamemory accuracy was positively associated with metamemory resolution, with a medium effect size. FDM accuracy was not associated with FDM resolution.

#### III. Awareness scores within task

FCAI pre- and post-awareness metrics (resolution and calibration) showed a strong, positive correlation with large effect sizes. Resolution and calibration were not associated on any of the three tasks.

#### IV. Awareness scores across task

**Fig 2:**
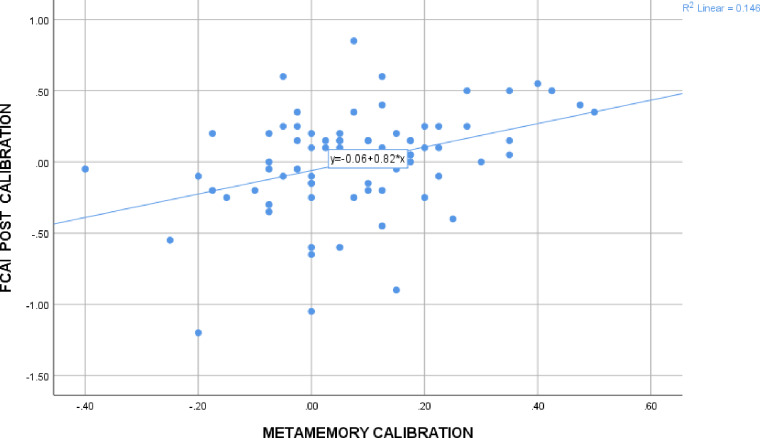


Metamemory calibration was positively associated with calibration on the FCAI (medium effect size; r range = .317-.359) but not DMC-F (see Fig 2). Calibration scores were positively associated across FDM tasks, with small to medium effect sizes; r range = .298-.354). None of the resolution scores were associated.

## Discussion

This is the first study to investigate and compare the robustness of different metrics for measuring FA, a relatively underexplored but increasingly important construct. Identifying an empirically-based FA measure is critical because impaired FA can lead to financially risky and damaging behaviors (e.g., overspending, getting scammed), especially in vulnerable populations such as older adults. Following established measurement frameworks from the metamemory literature, we examined the utility of two awareness metrics–calibration and resolution–using two different FDM tasks [26–28, 30]. Given that younger and older adults performed comparably on FDM and FA, data were combined across the two age groups. Neither FA metric was significantly associated with age, education or gender. However, important associations were found amongst the FA metrics and FDM that provide an initial guide for researchers, clinicians, and policy makers regarding how to best assess the construct of FA.

The primary finding of the current study was that FCAI calibration best met the a priori criteria we set for a robust FA metric. Specifically, we posited that the optimal FA metric would be: (1) associated with FDM itself, (2) associated with the corresponding FA metric on a separate FDM task, and (3) associated with performance on a metamemory task. Analysis of the calibration scores revealed that overconfidence on the FCAI was associated with lower levels of FDM, as well as overconfidence on a second FDM task, and overconfidence on the metamemory task. Although larger replication studies are required, FCAI calibration was the only FA metric of the four measured that met these criteria. Importantly, the association between overconfidence in FA and lower FDM scores is consistent with previous studies linking decreased FDM accuracy with impaired FA measured using informant report [6, 9] as well as the extant metamemory literature [31]. In contrast to the FCAI, overconfidence on the DMC-F was not associated with overconfidence on the metamemory task. The discrepancy in findings for these two FDM tasks likely reflects divergence in task characteristics, demands and components of the DMC-F versus the FCAI. Specifically, the DMC-F consists of items arranged in order of increasing difficulty and accurate performance requires that participants select a pre-determined response from the information displayed in a table. On the other hand, accurate performance on the FCAI requires the ability to perform tasks (e.g., writing a check) or generate responses to conceptual-level questions (for details see Methods section). In particular, the fact that the DMC-F consisted of only 6 items as compared to the 20-item FCAI may restrict calculation of robust FA scores. Indeed, it has been suggested that studies examining metamemory should use a large number of items (at least more than 15) in order to calculate more reliable scores [32–34]. In the current study, this aspect is captured by the lower internal consistency (.44) for the DMC-F compared to the FCAI (.71). However, we do acknowledge that the idea that 6 items are insufficient to calculate a robust FA score is speculative, and further studies are required to examine the validity of this notion.

Interestingly, resolution did not emerge as a robust FA metric for either FDM measure, relating neither to FDM performance nor to metamemory. One reason for the differential utility of calibration versus resolution as an awareness metric could be related to the structure of the FDM tasks. In traditional metamemory tasks, items within a task are generally similar (e.g., list of words, word-pairs, or sentences to be recalled) and may be repeated across multiple learning trials, task features which may both improve resolution among cognitively healthy individuals, and increase the reliability of the gamma statistic [33, 34]. In fact, it has been shown that with increased repetition of stimuli within a task, resolution improves reliably [35]. Similarly, repeated exposure to task structure has been shown to improve the accuracy of predictions [33]. The FCAI, used in the current study, consists of 20 varied items (e.g., writing a simulated check, reading a fake bill, etc.), perhaps challenging participants’ ability to carefully adjust their estimations from one item to the next. Assessment of FA may thus be most valid among a homogenous set of items with a clearly defined task structure. Researchers interested in exploring resolution related to FA may thus benefit from developing tasks that meet at least the following 2 criteria: (i) the task should consist of a relatively large number of items and, (ii) the task structure should involve items that are relatively similar and with varying difficulty levels to encourage the adjustment of predictions for performance.

The lack of a significant association between resolution and calibration for either of the FA tasks is not unexpected, and in fact has been found in previous metamemory studies, including this current study in which metamemory awareness metrics were not linked [23, 36]. Indeed, the two metrics measure different aspects of awareness—whereas resolution focuses on the relative congruence of the judgements and performance, calibration focuses on the absolute congruence of judgements and performance. One score can be fully intact despite impairment in the other. Although the manner in which these metacognitive processes become dissociated is not entirely clear, neuroimaging work has demonstrated that the metrics are associated with different brain regions [23, 24].

### Demographics, FDM and FA

The lack of age difference in FDM accuracy and FA metrics was interesting. One explanation for this finding may be related to the nature of FDM tasks which consist of items that are generally expected to be successfully completed by cognitively healthy, college-educated adults. In this context, both performance and confidence levels would be expected to be relatively high. Indeed, in the current study, the younger adults (mean age 41 years) and older adults (mean age 69 years) obtained scores near ceiling on both FDM tasks (see Table 1). Indeed, another study using an adapted version of FCAI in Netherlands also did not find a significant association between age and FDM in cognitively intact adults [37]. In the current study, therefore, it appears that the homogenous sample in the current study consisting of highly educated individuals may have attenuated the relationship among age and FDM and FA because of their high level of performance and confidence judgements. Regarding awareness, there are mixed findings in the literature as to whether age-differences are evident in calibration and resolution on metamemory tasks among the cognitively healthy [31]. For example, studies show that older adults tend toward underconfidence more so than younger adults on episodic metamemory tasks but are equally confident on semantic metamemory tasks that rely on prior knowledge [31]. Therefore, it has been proposed that age-differences in awareness metrics may be task-dependent. Indeed, all the participants in this study may have had prior knowledge and experience with the FDM tasks. In that sense, one may argue that the FDM tasks were more akin to semantic metamemory tasks. In fact, studies have found that level of education is associated with financial behaviors [38]. Our findings offer support to this link because the level of educational attainment was comparable across the age groups. Characterizing FA calibration across adulthood in future studies will shed light on whether overconfidence related to lowered FDM accuracy is a normative phenomenon. An important direction for future studies will be to track the association between FDM and FA longitudinally as adults age and as cognitive deficits become evident, and examine if, when, and how changes in these constructs emerge, and how they influence real-world behaviors.

The finding that years of education and gender were not associated with any of the FDM or FA metrics is somewhat surprising. Studies have typically found that higher level of education, increased gross annual income, and being male is associated with better FDM and higher levels of confidence [37, 38]. The absence of a significant association in the current study therefore merits attention. It is plausible that the demographic characteristics of the current sample may play a role in attenuating such effects. Specifically, participants were on average college educated (mean = 15 years), and both males and females had comparable levels of education (*t* = .52, *p* = .60, *d* = -0.11). Although replication is required, it is possible that high levels of education may reduce or eliminate gender effects on FDM and FA. However, the study by Bangma et al. (2017) also had participants with average college education, yet education was associated with FDM. Interestingly, in their study, education was also found to be associated with intuitive financial decisional style. It is therefore possible that other factors associated with decisional style, cultural influences, quality of education and the type of experience with everyday finances may influence the relation between education and FDM.

To conclude, current findings offer initial guidance to researchers and clinicians regarding how to measure FA, and to public policy makers regarding how to incorporate FA into models of FDM and design strategies for preserving FDM in the aging population. In the context of financial education, the association between the FDM and FA underscores the value of teaching individuals to understand and monitor their financial habits while emphasizing the common pitfalls associated with under- and over-confidence. There are, however, several limitations to this study. First, as this is a cross-sectional study, inferences about causality cannot be drawn; future studies should be designed to longitudinally track FDM with FA. Moreover, although the association between FA and FDM underscores that FA is likely a construct that is related to, but independent, from FDM itself, there remains much to be examined in terms of how FA is associated with various aspects of cognition. It is also important to bear in mind, that the current study used prospective and retrospective judgements that varied across the tasks. However, in the current study, the FCAI prospective and retrospective judgements were highly correlated across awareness metrics (see Table 3). Previous studies have also found that metamemory judgements do not significantly differ in prospective vs. retrospective conditions (Chapman et al., 2018; Cosentino et al. 2011). Based on these findings, the prospective and retrospective judgements in the DMC-F task will not be expected to differ substantially in healthy controls. However, this speculation needs to be empirically tested in future studies. Lastly, the performance on the FDM tasks was characterized by limited variability due to the task demands and because of the highly educated nature of our sample. In order to generalize these findings, using more challenging FDM tasks across individuals with varying levels of education is required. Nevertheless, this is the first study to formally investigate the properties of FA in the context of aging, providing a platform for future research to better develop FDM and FA tasks. While designing FA tasks, it will be critical to match the accuracy and confidence ratings for the FDM task from the outset to enable a straightforward calculation of various awareness metrics. It is now recognized that FDM, used synonymously with other terms like financial capacity, financial management, financial capability, or financial competency is a broad construct that subsumes multiple dimensions [3, 27, 30, 39–42]. It is thus possible that FA may vary across these different dimensions and the tasks that measure them. Therefore, it is important to measure FA using more than one task, and to understand the interrelation between the different FA scores generated. Additional work is needed to understand the extent to which awareness varies across financial activities (e.g., paying bills, reviewing credit card statements, investing in mutual funds), or whether they are linked by a higher order or global FA construct.

## Supporting information

S1 Text (DOCX)

S1 Data SPSS data. (SAV)
