# Supplementary material for: Self-awareness for financial decision-making abilities in healthy adults
Source: PLoS One. 2020 Jul 2;15(7):e0235558. doi: 10.1371/journal.pone.0235558 (PMC7332073; doi:10.1371/journal.pone.0235558)
Supplement: S1 Text — (DOCX) [file pone.0235558.s001.docx]

**SUPPLEMENTARY TEXT**

| **Table 1: Association among performance measures after adjusting for education and gender.** | | |
| --- | --- | --- |
| **Spearman's rho (*p*)** | | |
|  | FCAI | DMC-F |
| Memory | .20 (.07) | .20 (.07) |
| FCAI | - | .39 (.00) |

**Procedures for Recoding DMC-F and FCAI**

In order to calculate metacognitive scores, the original coding systems for accuracy and confidence required recoding in three ways. For any study, in order to generate calibration scores for which 0 is perfectly calibrated, < 0 is underconfident, and > 0 is overconfident, the coding systems must have two characteristics. First, higher confidence must be represented with higher numerical ratings; second, such ratings must be on the same scale as accuracy. Additionally, in the current study, we re-coded data based on their distribution; specifically, certain confidence ratings are endorsed very infrequently. For example, the “unsure” and “not at all confident” ratings on the DMC-F and the FCAI. As such, we collapsed these two confidence ratings into one category, and obtained three confidence levels (1, 2, and 3) which were numerically coded to 0, 0.5, and 1 to match the accuracy levels.

Finally, certain data were recoded based on the characteristics of the tests themselves. Specifically, accuracy scores on the FCAI have subtle differences (for example, 4 = Complete Understanding and 3 = Adequate Understanding).  For the purposes of creating metacognitive scores, requiring individuals to discriminate between their performance at the level of 4 versus 3 is not as critical as their ability to discriminate between these combined levels versus items on which they received a 2 (Partial Understanding). In fact, it was our expectation that requiring participants to provide self-ratings that would discriminate between an item for which they scored a 3 versus an item on which they scored a 4 could result in less reliable metacognitive scores. Rather, our goal was to determine whether people could accurately discriminate between broader categories of performance.  Therefore, accuracy data were collapsed across accuracy levels.

The specific tests were recoded as follows:

**1. DMC-F:**Accuracy for each item was coded as binary, with 0 = inaccurate and 1 = accurate whereas confidence ratings ranged from 1 to 4 (1 = *very confident, 2 = somewhat confident, 3= unsure, 4 =* *not at all confident*). To address the issues above, the “unsure” and “not at all confident” ratings were first collapsed due to infrequent endorsement (see Supplementary Figure 1). The three remaining levels (1, 2, and 3) were then recoded to 0, 0.5, and 1 to match the range of the accuracy scale, and reversed such that higher values represented increasing confidence. 

 **2. FCAI:** Accuracy for each item was originally coded on a 5-point scale (0 = No Understanding, 1 = Minimal Understanding, 2 = Partial Understanding, 3 = Adequate Understanding, and 4 = Complete Understanding). Accuracy scores were collapsed into a 3-point scale in accord with clinical utility as follows: 0 and 1 were collapsed into one category (No/Minimal Understanding), while 3 and 4 were collapsed into one category (Adequate/Complete Understanding). With regard to confidence, as described for the DMC-F, ratings were originally made on a 4-point scale and collapsed to a 3-point scale (see Supplementary Figure 2). The three remaining levels (1, 2, and 3) matched the range of the accuracy scale, but were reversed such that higher values represented increasing confidence.

**Supplementary Figure 1:** Mean Frequency of Endorsed Confidence Levels for DMC-F

**Supplementary Figure 2:** Mean Frequency of Endorsed Confidence Levels for FCAI
